# Supplementary material for: Depth of Response to Intensive Chemotherapy Has Significant Prognostic Value among Acute Myeloid Leukemia (AML) Patients Undergoing Allogeneic Hematopoietic Stem-Cell Transplantation with Intermediate or Adverse Risk at Diagnosis Compared to At-Risk Group According to European Leukemia Net 2017 Risk Stratification
Source: Cancers (Basel). 2022 Jun 29;14(13):3199. doi: 10.3390/cancers14133199 (PMC9265052; doi:10.3390/cancers14133199)
Supplement: Supplementary file 1 [file cancers-14-03199-s001.zip › cancers-1766067-supplementary.pdf]

Supplementary Information

		Univariate		Multivariate	
**End Point and Variables (Total n=174)**	**N**	**HR (95% CI)**	**p-values**	**HR (95% CI)**	**p-values**
**OS**					
**Cytogenetic adverse risk group**	32	1.21 (0.69-2.15)	0.513		
***FLT3*-ITD mutation**	30	2.01 (1.16-3.48)	0.019	1.88(0.97-3.63)	0.061
***NPM* mutation**	16	1.83(0.91-3.70)	0.116	1.18(0.51-2.74)	0.698
***RUNX1* mutation**	23	0.45 (0.18-1.11)	0.084		
***ASXL1* mutation**	14	0.26 (0.06-1.06)	0.018		
***TP53* mutation**	6	1.88 (0.68-5.16)	0.263		
**RFS**					
**Cytogenetic adverse risk group**	32	1.36(0.82-2.28)	0.238		
***FLT3*-ITD mutation**	30	1.92(1.15-3.20)	0.013	2.07(1.23-3.47)	0.017
***NPM* mutation**	16	1.51(0.75-3.01)	0.272		
***RUNX1* mutation**	23	0.43(0.19-0.99)	0.048	0.50(0.21-1.20)	0.119
***ASXL1* mutation**	14	0.43(0.16-1.17)	0.060	0.59(0.21-1.70)	0.332
***TP53* mutation**	6	2.06(0.83-5.10)	0.157		
**CIR**					
**Cytogenetic adverse risk group**	32	2.46(1.38-4.37)	0.002	2.66(1.41-5.01)	0.002
***FLT3*-ITD mutation**	30	2.01(1.01-4.02)	0.048	2.65(1.26-5.56)	0.010
***NPM* mutation**	16	1.49(0.19-2.34)	0.532		
***RUNX1* mutation**	23	0.96(0.40-2.31)	0.924		
***ASXL1* mutation**	14	0.43(0.11-1.63)	0.215		
***TP53* mutation**	6	3.54(1.27-9.87)	0.015	3.10(1.25-7.66)	0.014
**NRM**					
**Cytogenetic adverse risk group**	32	0.39(0.12-1.29)	0.123		
***FLT3*-ITD mutation**	30	1.22(0.54-2.77)	0.632		
***NPM* mutation**	16	2.32(0.97-5.56)	0.059		
***RUNX1* mutation**	23	0.54(0.13-2.24)	0.390		

***ASXL1* mutation**	14	0.64(0.16-2.50)	0.520
***TP53* mutation**	6	0.74(0.12-4.59)	0.7485

N, number of patients; CI, confidence interval; HR, hazard ratio; OS, overall survival; RFS, relapse-free survival; CIR, cumulative incidence of relapse; NRM, non-relapse mortality

Supplementary Table S1. Univariate and Multivariate analysis for survival outcomes related to ELN2017

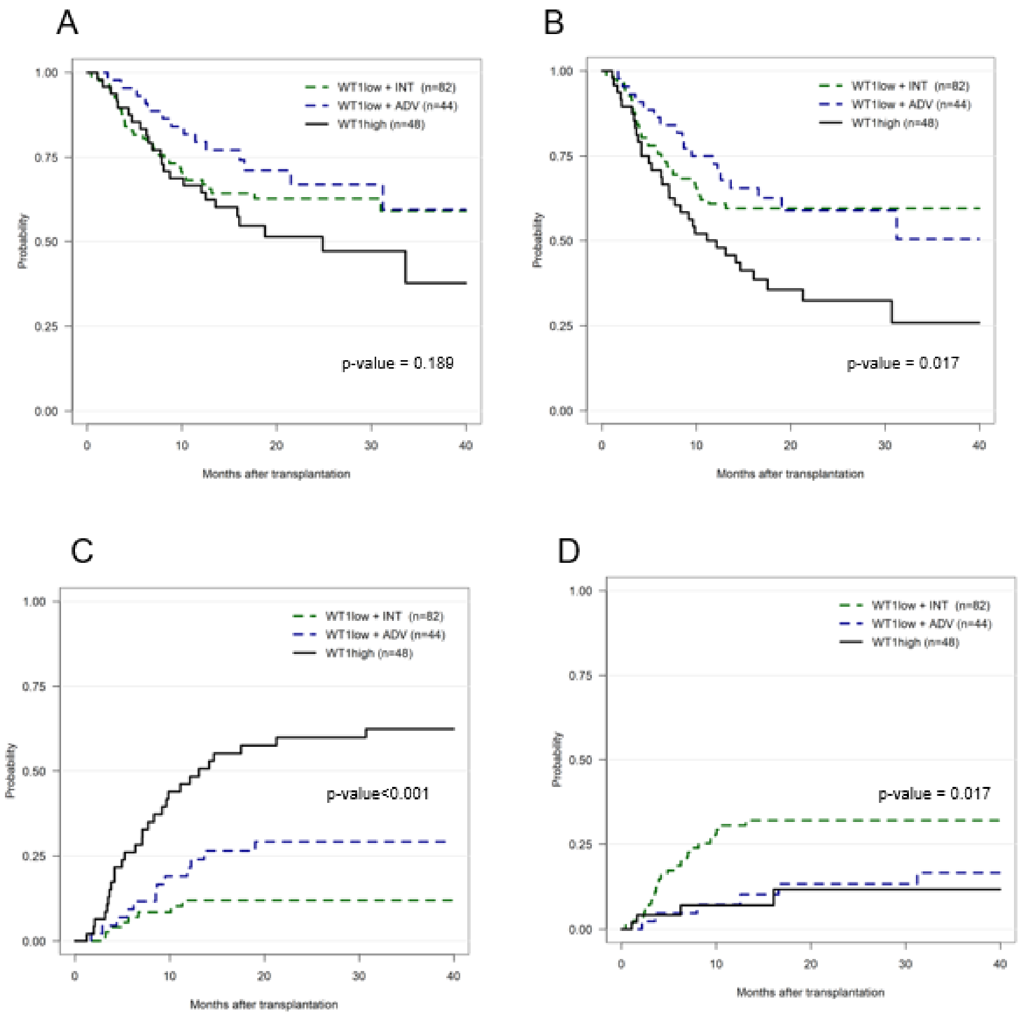

Supplementary Figure S1. Prognostic significance of *WT1* level in patients with AML who undergo allogeneic HSCT. Appliance to the 2 risk groups according to *WT1* level predicted OS (A), RFS (B), CIR (C), and NRM (D).

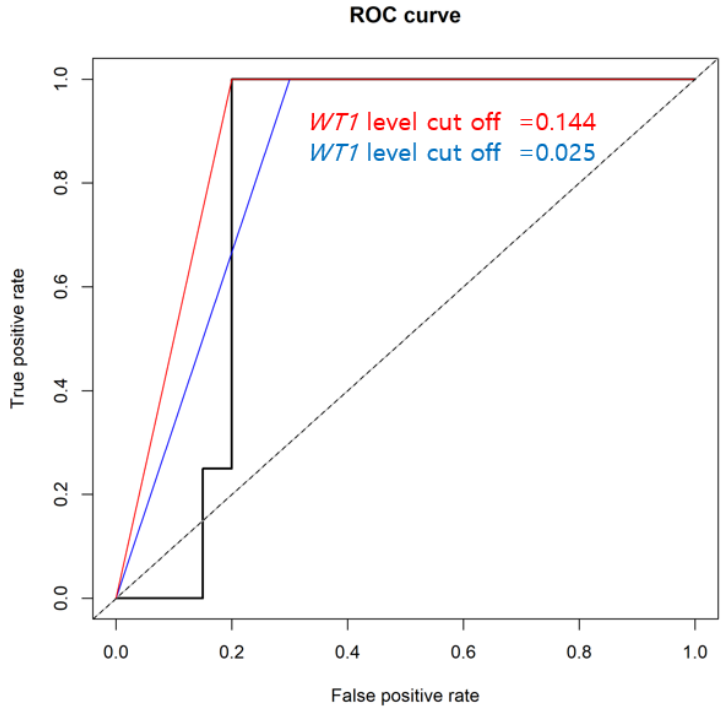

Supplementary Figure S2. Receiver operating characteristics (ROC) analysis with *WT1* level cut-off and *FLT3*-ITD MRD positivity Pre-HSCT.
